# An Unpardonable Liberty

**Published:** 1892-04

**Authors:** 


### AN UNPARDONABLE LIBERTY.

"Dr." T. H. Bragg an erstwhile homeopathic physician of Austin having engaged in the Keely Institute whisky cure business takes the liberty (!) of referring in a circular to our State Health Officer without permission or without even his knowledge. This bold and presumptuous act his former partner Dr. C. E. Fisher, editor of the *Southern Journal of Homeopathy*, in an editorial denounces in the strongest language, and says Dr. Swearingen is too clean a man to lend his name to any such connection.

We could not have said it better. Of course Dr. Swearingen repudiates the use of his name as unauthorized and an unwarranted liberty; but he is too "clean" a man to touch the subject, even in the way of a denial in print; it is too small a matter for him to trouble himself about.

